# The efficacy and safety of micropulse transscleral laser treatment in glaucoma: a systematic review and meta-analysis

**DOI:** 10.1186/s12886-023-03017-w

**Published:** 2023-06-12

**Authors:** Qiying Ling, Ziyan Cai, Xinyue Zhang, Xuanchu Duan

**Affiliations:** 1 Aier Eye Hospital, Jinan University, Guangzhou, Guangdong Province 510071 China; 2 Changsha Aier Eye Hospital, Changsha, Hunan Province China; 3 Department of Ophthalmology, The Second Xiangya Hospital of Central South University, Changsha, Hunan Province China; 4 Glaucoma Institute, Changsha Aier Eye Hospital, Changsha, Hunan Province China

**Keywords:** Micropulse transscleral laser treatment, Continuous wave transscleral cyclophotocoagulation, Glaucoma, Efficacy, Safety, Meta-analysis

## Abstract

**Objective:**

Micropulse transscleral laser treatment (mTLT) is the latest alternative intraocular pressure (IOP) lowering approach for glaucoma patients. This meta-analysis aims to evaluate the efficacy and safety of mTLT and continuous wave transscleral cyclophotocoagulation (CW-TSCPC) for the treatment of glaucoma.

**Methods:**

We searched the PubMed, Embase, and Cochrane Library of Systematic Reviews databases from January 2000 to July 2022 to identify studies that, evaluated the efficacy and safety of mTLT in glaucoma. There were no restrictions regarding study type, patient age, or type of glaucoma. We analysed the reduction in IOP and the number of anti-glaucoma medications (NOAM), retreatment rates, and complications between mTLT and CW-TSCPC treatment. Publication bias was conducted for evaluating bias. This systematic review followed the Preferred Reporting Items for Systematic Reviews and Meta-analyses (PRISMA 2020) reporting guideline.

**Results:**

We identified 6 eligible studies of which only 2 RCTs and 386 participants with various types of glaucoma at different stages were ultimately included. The results revealed significant IOP decreases after mTLT up to 12 months and significant NOAM reductions at 1 month (WMD=-0.30, 95% CI -0.54 to 0.06), and 3 months (WMD=-0.39, 95% CI -0.64 to 0.14) in mTLT compared to CW-TSCPC. Moreover, the retreatment rates (Log OR=-1.00, 95% CI -1.71 to -0.28), hypotony (Log OR=-1.21, 95% CI -2.26 to -0.16), prolonged inflammation or uveitis (Log OR=-1.63, 95% CI -2.85 to -0.41), and worsening of visual acuity (Log OR=-1.13, 95% CI -2.19 to 0.06) occurred less frequently after mTLT.

**Conclusion:**

Our results demonstrated that mTLT could lower the IOP until 12 months after treatment. mTLT seems to have a lower risk of retreatment after the first procedure, and mTLT is superior to CW-TSCPC with respect to safety. Studies with longer follow-up durations and larger sample sizes are necessary in the future.

**Trial registration number:**

INPLASY202290120.

**Supplementary Information:**

The online version contains supplementary material available at 10.1186/s12886-023-03017-w.

## Introduction

Glaucoma is a threat to visual health around the world and is characterized by irreversible optic neuropathy and highly elevated intraocular pressure (IOP). The worldwide prevalence of glaucoma among individuals aged 40–80 years is estimated to be 3.54%. By 2040, the number of people suffering from glaucoma is expected to rise to 111.8 million globally [1]. IOP-lowering treatment remains a crucial procedure for glaucomatous patients. Transscleral laser treatment can reduce aqueous humour production through ciliary body destruction, thereby reducing IOP and protecting the optic nerve. Commonly applied diode laser treatments include micropulse transscleral laser treatment (mTLT) and continuous wave transscleral cyclophotocoagulation (CW-TSCPC). In traditional treatment, CW-TSCPC is regarded as the last resort for advanced stage of refractory glaucoma even after the administration of various anti-glaucoma medications, laser therapy and other surgical treatments. However, the severe complications (e.g., worsening of visual acuity, hypotony, inflammation) associated with CW-TSCPC limit its clinical applications. On the other hand, mTLT has been gradually developed into a new alternative laser modality, with the superiority of relatively fewer complications and a decent efficacy [2]. Most importantly, mTLT expands the indications of cyclophotocoagulation, and it is not only applicable in the advanced stage of glaucoma [3]. However, the efficacy and safety of mTLT varies across studies, and no relevant systematic review has been conducted.

## Methods

### Study protocol

This study protocol was registered in the INPLASY (INPLASY202290120) database. This systematic review was carried out in accordance with the Preferred Reporting Items for Systematic Reviews and Meta-Analyses recommendations.

### Search strategy

The PubMed, Embase, and Cochrane Library of Systematic Reviews electronic databases were searched from January 1, 2000 to July 29, 2022 to identify all clinical studies examining the use of mTLT for glaucoma. In addition to using Medical Subject Headings (MeSH) terms (‘Glaucoma’, and ‘Laser Coagulation’), the commercial name and other text terms were also reviewed.

The search strategy for PubMed was as follows: (((((((((((((((((((((Thermocoagulation, Laser[Title/Abstract]) OR (Coagulation, Laser[Title/Abstract])) OR (Coagulations, Laser[Title/Abstract])) OR (Laser Coagulations[Title/Abstract])) OR (Laser Thermocoagulation[Title/Abstract])) OR (Laser Thermocoagulations[Title/Abstract])) OR (Thermocoagulations, Laser[Title/Abstract])) OR (Transscleral cyclophotocoagulation with MicroPulse laser[Title/Abstract])) OR (MicroPulse™ transscleral cyclophotocoagulation[Title/Abstract])) OR (Micropulse Transscleral Diode Cyclophotocoagulation[Title/Abstract])) OR (Micropulse Transscleral Diode Laser Cyclophotocoagulation[Title/Abstract])) OR (MicroPulse® transscleral laser therapy[Title/Abstract])) OR (micropulse cyclophotocoagulation[Title/Abstract])) OR (micropulse transscleral laser cyclophotocoagulation[Title/Abstract])) OR (Micropulse Diode Transscleral Cyclophotocoagulation[Title/Abstract])) OR (Micropulse Laser Transscleral Cyclophotocoagulation[Title/Abstract])) OR (Micropulse Trans-scleral Cyclophotocoagulation[Title/Abstract])) OR (Micropulse trans-scleral diode laser cyclophotocoagulation[Title/Abstract])) OR (Micropulse Transscleral Laser Treatment[Title/Abstract])) OR (micro-pulse transscleral cyclophotocoagulation[Title/Abstract]) OR (Laser Coagulation[mh]) AND ((Glaucomas[Title/Abstract]) OR (Glaucoma[mh])) AND (2000/1:2022/7[pdat]). Additionally, we manually searched the references of the retrieved articles.

### Study selection

#### Inclusion criteria

- All study types except case reports or reviews (e.g. randomized controlled trials (RCTs), retrospective or prospective cohort studies, and case-control studies);
- A comparative study of mTLT and other cyclodestructive procedures;
- Research subject: all kinds of glaucoma;
- Research content: the efficacy and safety of mTLT;
- Without gender, race, age, and surgical history restrictions;
- Without the laser energy and duration restrictions;

#### Exclusion criteria

- Animal research, case report, review, clinical trials without result, and conference abstract.
- Duplicate publication.
- Publications not in English.
- Combined with other surgeries.
- Without a control group.

Two authors (Q.L. and Z.C.) screened the titles and abstracts of all retrieved studies according to the inclusion criteria and eliminated studies based on the exclusion criteria. And a third investigator (X.Z.) was involved in resolving disagreements.

### Data extraction and quality assessment

Two investigators (Q.L. and Z.C.) independently assessed trial eligibility and extracted data. The following data were extracted from each study: authors, publication year, study design (RCT, prospective study and respective study), interventions, participants’ characteristics (number, age, type of glaucoma, baseline IOP), the characteristics of the mTLT and CW-TSCPC, follow-up duration, and outcome parameters. The revised Cochrane risk of bias, version 2 (RoB 2) tool [4] for RCTs and Newcastle–Ottawa Scale [5] for nonrandomized studies were applied to quality assessment.

### Outcome measures

Therapeutic efficacy included IOP reduction ( IOPR)and the reduction in number of the anti-glaucoma medications (NOAMR) from the baseline to endpoints at various follow-up visits, as well as retreatment rates. The retreatment rates in our analysis were defined as the number of patients requiring repeated laser or surgical treatment after the first laser procedure. The therapeutic safety was evaluated by the complications including choroidal detachment, hyphema, hypotony, pain after laser, phthisis bulbi, prolonged inflammation or uveitis, pupil distortion, scleral thinning, and worsening of visual acuity.

When some studies only reported the baseline and endpoint IOPs, their IOPR and standard deviation (SD) of the IOPR (SD_IOPR_) were calculated using the following formulas [6]:

IOPR = IOP_baseline_ − IOP_endpoint_.

SD_IOPR_ = (SD^2^_baseline_ + SD^2^_endpoint_ − SD_baseline_ + SD_endpoint_)^1/2^.

### Statistical analysis

The analysis was conducted by Stata version 17 software. In this study, continuous outcomes (IOP, IOPR, number of the anti-glaucoma medications (NOAM)and NOAMR) were expressed as weighted mean differences (WMDs), while dichotomous outcomes (retreatment rates and complication) were expressed as Log odds ratios (Log OR). The I^2^ test was used to determine the magnitude of heterogeneity between publications. Heterogeneity was categorized as high (I^2^ > 75%), moderate (75% >I^2^ > 25%), or mild (I^2^ < 25%). When I^2^ < 50%, the fixed effects model was chosen, otherwise, the random effects model was suitable.

We used the Egger’s regression test and Begg’s test [7] to assess publication bias, where a significance level of P < 0.05 was considered statistically significant. If publication bias was detected, we applied the trim-and-fill method to adjust for the bias. Subsequently, we replicated the funnel plot by adding the ‘missing’ counterparts around the adjusted summary estimate. In addition, the number of included studies limited the sensitivity analysis.

### Patient and public involvement

Patients and the public were not involved in this study.

## Results

### Study selection

**Fig. 1:**
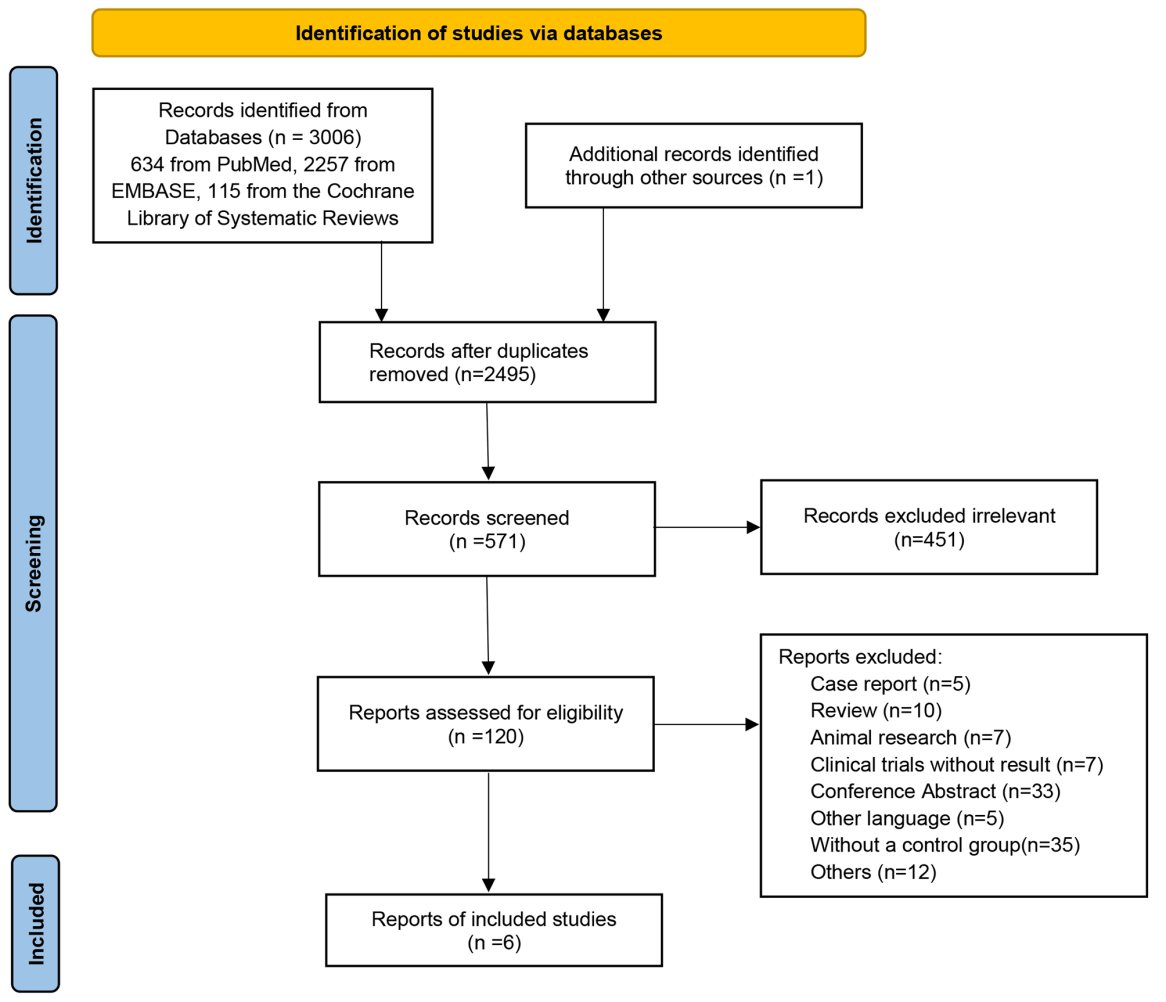
Flow diagram of the selection process. Initially, 3007 documents were retrieved, of which 2495 remained after duplicates were removed. After screening the titles and abstracts, 451 irrelevant studies, 5 case reports, 10 reviews, 7 animal studies, 7 clinical trials without results, 33 conference abstracts, 5 studies in other languages, 35 studies lacking a control group, and 12 studies with other reasons were excluded. Ultimately, 6 documents were included in this meta-analysis

There were 3007 documents retrieved initially and 2495 remained after duplicates were removed. After screening the titles and abstracts, 451 irrelevant studies were excluded. Five case reports, 10 reviews, 7 animal studies, 7 clinical trials without results, 33 conference abstracts, 5 studies in other languages, 35 studies without a control group, and 12 studies with other reasons were excluded. Finally, 6 documents, including 2 RCTs, 2 retrospective studies, and 2 prospective studies, were selected based on the inclusion and exclusion criteria in this meta-analysis (shown in Fig. 1). All the comparisons were between mTLT and CW-TSCPC.

### Patient characteristics and quality assessment

**Fig. 2:**
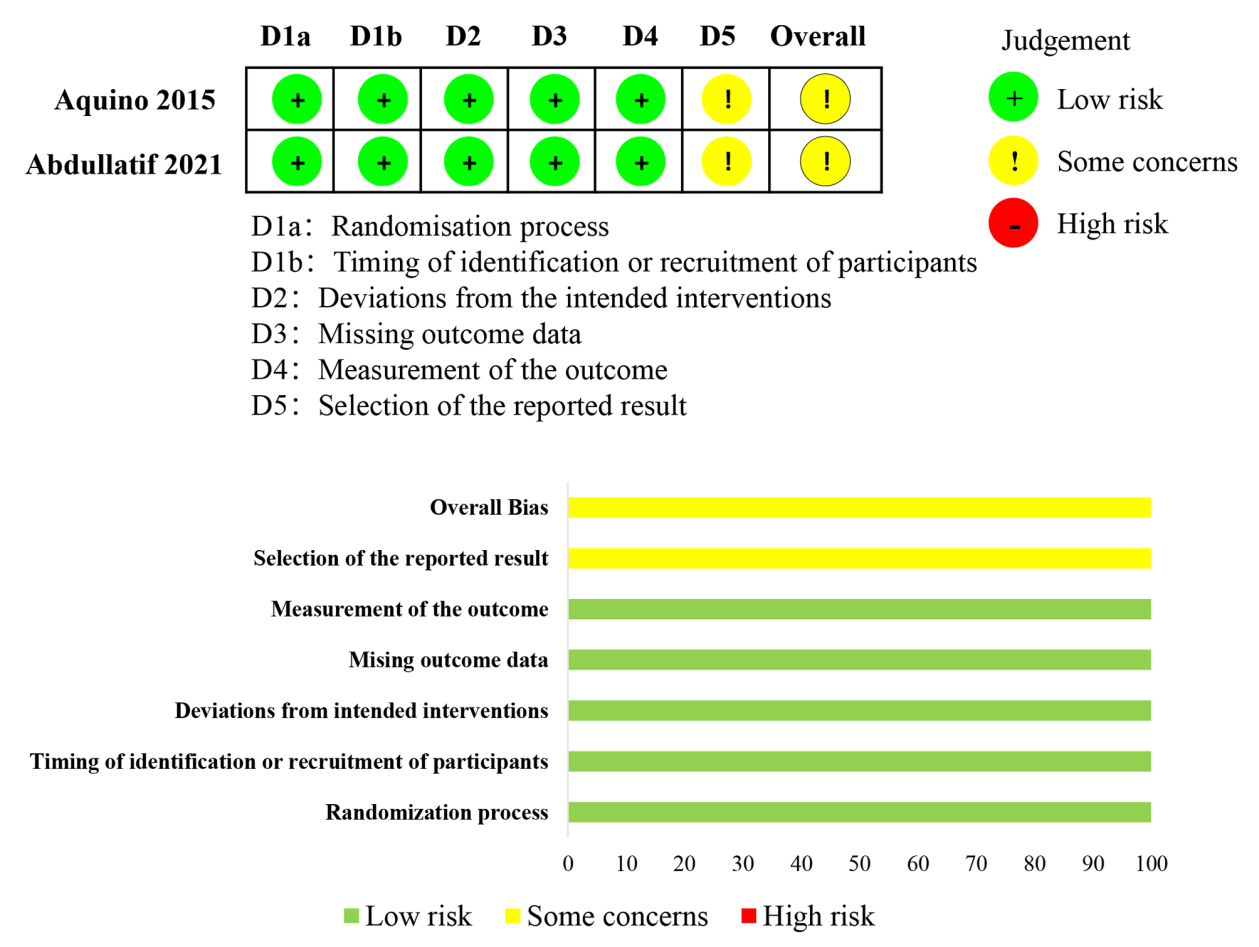
Summary of the revised Cochrane risk of bias, version 2. In these two RCTs, only “Selection of the reported result” was judged as some concerns, while the other five items were considered low risk

A total of 137 patients treated by mTLT and 249 patients treated by CW-TSCPC were included, in multiple ages (child, middle-aged and elderly), with different types of glaucoma at various stages, and with or without surgery history. The follow-up ranged from 6 to 18 months. mTLT and CW-TSCPC laser energy ranged from 2 to 2.8 W and 1.25 to 2.3 W respectively, while their durations were from 100 to 200 s and from 36 to 84 s respectively. Moreover, mTLT had with a duty cycle of 31.3%. In Table 1, all included studies and the characteristics of glaucoma patients are described. The quality assessment of all included studies is shown as Fig. 2; Table 2.

**Table 1:** The characteristics of the included studies in this meta-analysis

Author/year	Abdelrahman 2018 [21]	Abdullatif 2021 [28]	Aquino 2015 [44]	Bernardi 2022 [45]	Fili 2022 [46]	Zemba 2022 [18]
**Research type**	Prospective study	RCT	RCT	Retrospective study	Prospective study	Retrospective study
**Age (mean ± SD)** **(mTLT / CW-TSCPC)**	67.8 ± 48 / 61.3 ± 38.3 months	48.6 ± 6.6 / 53 ± 10.6 years	63.50 (54.75,74) / 66 (55, 72.75) years	68.9 ± 13.3 / 73.1 ± 15.5 years	77.77 ± 10.97 years	55.6 (range, 44‑79) / 58.1 (range, 32‑87) years
**Type of glaucoma**	PCG, Aphakia/pseudophakia Aniridia glaucoma, Peter’s anomaly, Microspherophaki, Sturge Weber glaucoma	open-angle glaucoma	POAG, PACG, NVG, Silicone oil, Aphaki, Traumatic glaucoma	POAG, ACG, PEXG, PDG, Aphakic glaucoma, OHT, Others	POAG, PEXG	NVG
**Characteristics of mTLT (power, duration)**	2000 mW, a duty cycle of 31.3%, 100 to 120 s	2000 mW, a duty cycle of 31.3%, 200 s	2000 mW, a duty cycle of 31.3%, 100 s	2000 mW, a duty cycle of 31.3%, 160 s	2500 mW, a duty cycle of 31.3%, >160 s	2000 mW, a duty cycle of 31.3%, 180 s
**Characteristics of CW-TSCPC (power, duration)**	1.5 W, 45 s	1.5–2 W, 40–56 s	1.5–2 W, 40–56 s	2 W, 37.5 s	2–2.3 W, 36 s	1.25 W, 80–84 s
**No. of eyes (mTLT / CW-TSCPC)**	17 / 28	10 / 10	24 / 24	47 / 150	15 / 15	24 / 22
**Duration of follow-up (months)**	6	6	18	12	12	12
**Baseline IOP (mmHg, mean ± SD)(mTLT / CW-TSCPC)**	28.3 ± 8.2 / 27.5 ± 6.1	18.7 ± 2.9 / 19.8 ± 5.0	36.5 (29.5, 56.5) / 35.0 (29.5, 46.5)	22.0 ± 7.2 / 28.3 ± 12.3	17.75 ± 6.92 / 19.13 ± 7.27	34.7 ± 10.3 / 36.0 ± 13.2
**IOP reduction at the last visit (%) (mTLT / CW-TSCPC)**	63 ± 28 / 67 ± 25	23.9 ± 6.1 / 31.5 ± 25.3	-	31.1 / 43.7	-	23.0 / 33.6
**Success rates at the last visit (%) (mTLT / CW-TSCPC)**	71 / 46	60 / 50	52 / 30	87.5 / 88.6	60 / 20	33.3 / 54.5

RCT: randomized controlled trial; mTLT: micropulse transscleral laser treatment; CW-TSCPC: continuous wave transscleral cyclophotocoagulation; PCG: primary congenital glaucoma; POAG: primary open angle glaucoma; PACG: primary angle-closure glaucoma; NVG: neovascular glaucoma; ACG: angle-closure glaucoma; PEXG: pseudoexfoliative glaucoma; PDG: Pigment dispersion glaucoma; OHT: ocular hypertension

**Table 2:** Quality assessment for cohort studies [5]

Study	Selection					Comparability			Outcome			Quality score
	Outcomes not present pre-study	Representative treatment group	Representative reference group	Ascertain of exposure		Comparable for 1,2,3	Comparable for 4,5,6		Assessment of outcome	Adequate follow-up duration	Adequate follow-up cohorts	
**Abdelrahman 2018**[21]	*			*		1,2,3	4,5,6		*	*	*	11
**Bernardi 2022**[45]	*			*		1, 2,3	6		*	*	*	9
**Fili 2022**[46]	*			*			5,6		*	*	*	7
**Zemba 2022**[18]	*			*		1,2,3	4,5,6		*	*	*	11

1.Age 2. Sex 3. Diagnosis 4. Previous surgery 5. Initial intraocular pressure 6. Initial number of anti-glaucoma medicine

*means the quality requirements of the items were met

### Therapeutic efficacy assessments

#### IOP reduction

**Fig. 3:**
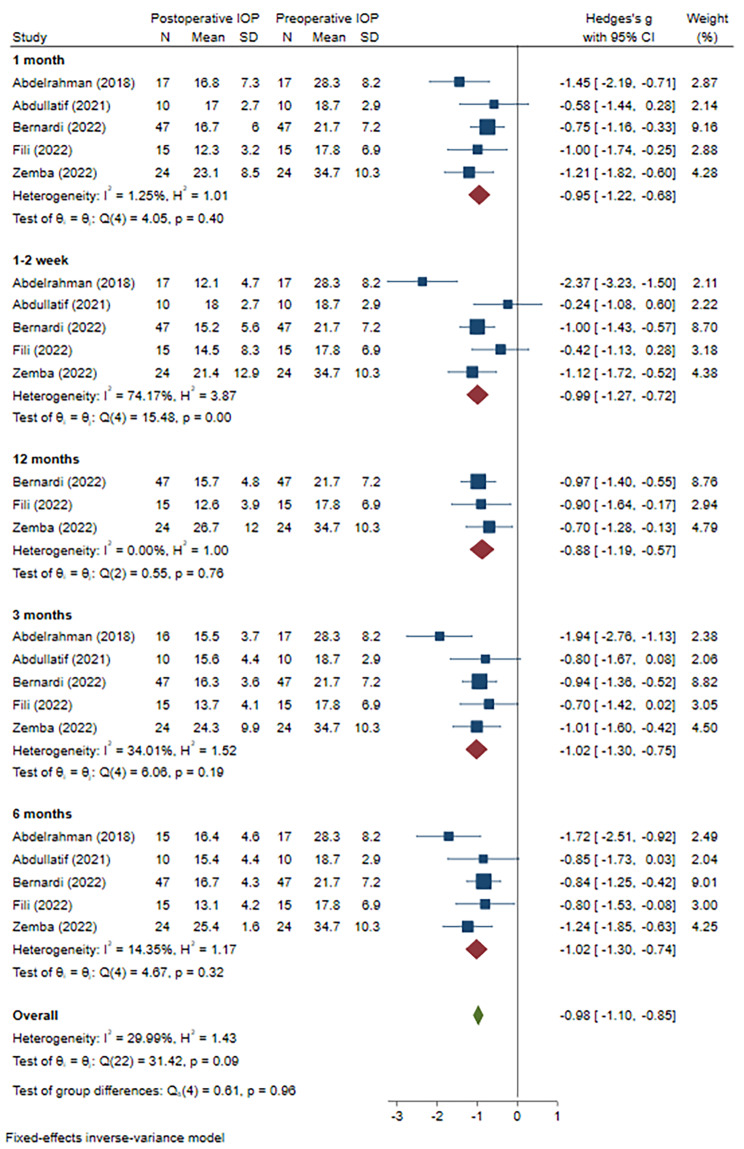
Meta-analysis of post- and pre-operative IOP in mTLT at various visits. A total of 103 patients treated with mTLT were included. The IOP was significantly reduced at 1, 3, 6, and 12 months (WMD=-0.95, 95% CI -1.22 to -0.68; WMD=-1.02, 95% CI -1.30 to -0.75; WMD=-1.02, 95% CI -1.30 to -0.74; WMD=-0.88, 95% CI -1.19 to -0.57, respectively)after mTLT.

**Fig. 4:**
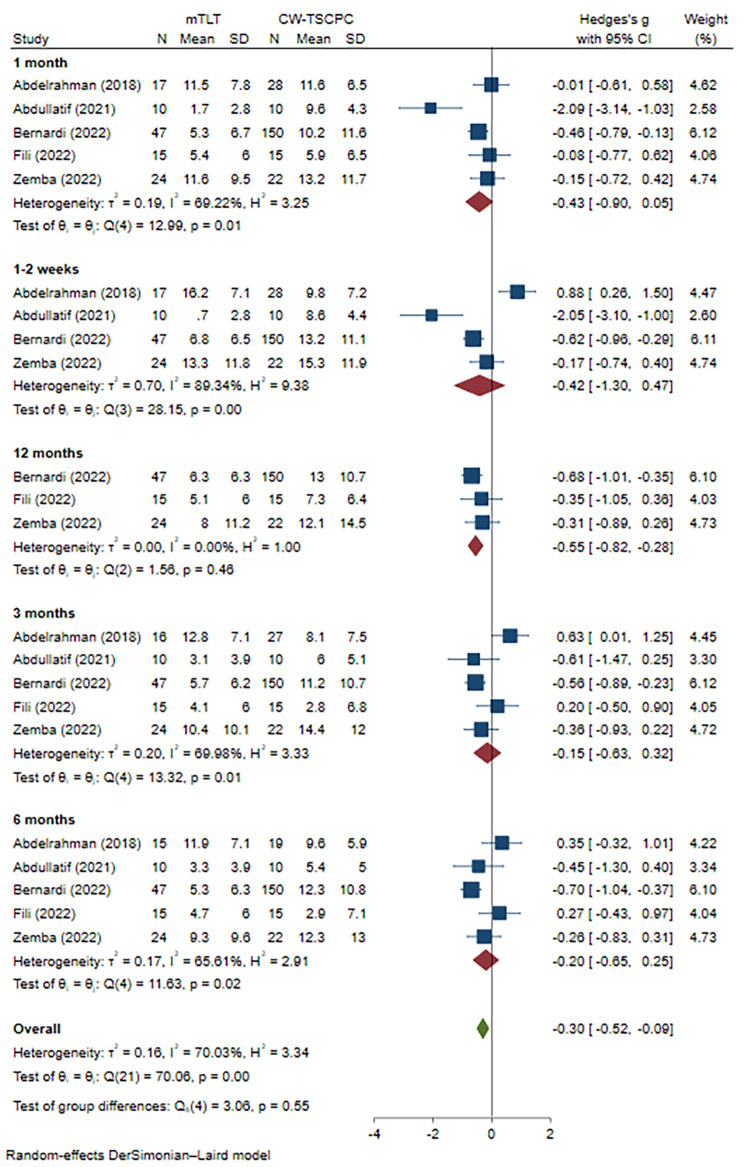
Meta-analysis of mTLT compared to CW-TSCPC in IOP reduction at various follow-up visits. A total of 113 patients treated with mTLT and 225 patients treated with CW-TSCPC were included. No significant difference was observed at 1–2 weeks, 1 month, 3 months, and 6 months after the operation, but there was with high heterogeneity. At 12 months, the IOP reduction in mTLT was inferior to that in CW-TSCPC (WMD=-0.55, 95% CI -0.82 to -0.28)

Due to the IOP changes in one article being presented as medians and quartiles, only 5 publications were included to evaluate the effect on IOP reduction finally. For a better evaluation, we adopted a subanalysis based on the follow-up visits. First, we compared the IOP values before and after the mTLT procedure. The heterogeneity was low (I^2^ = 1.25%, P = 0.40 at 1 month, I^2^ = 34.01%, P = 0.19 at 3 months, I^2^ = 14.35%, P = 0.32 at 6 months, and I^2^ = 0.00%, P = 0.76 at 12 months, respectively), and the results showed that under the anti-glaucoma medications, mTLT could effectively lower IOP till 12 months (WMD=-0.95, 95% CI -1.22 to -0.68 at 1 month, WMD=-1.02, 95% CI -1.30 to -0.75 at 3months, WMD=-1.02, 95% CI -1.30 to -0.74 at 6 months, and WMD=-0.88, 95% CI -1.19 to -0.57 at 12 months, respectively) **(**Fig. 3**)**. Then, we compared the baseline IOP between the mTLT and CW-TSCPC groups. The preoperative IOP in mTLT group was mildly lower than that in the CW-TSCPC group (WMD=-0.31, 95% CI -0.55 to -0.08), and there was heterogeneity among these studies (I^2^ = 14.84%, P = 0.01) **(Supplement** Fig. 1**)**. For the IOP reductions at each visit, no significant difference was observed at 1–2 weeks, 1, 3, and 6 months postoperatively (WMD=-0.42, 95% CI -1.30 to 0.47; WMD=-0.43, 95% CI -0.90 to 0.05; WMD=-0.15, 95% CI -0.63 to 0.32; and WMD=-0.20, 95% CI -0.65 to 0.25, respectively), but all with a high heterogeneity (I^2^ = 89.34%, P = 0.00; I^2^ = 69.22%, P = 0.01;I^2^ = 69.98%, P = 0.01 I^2^ = 65.61%, P = 0.02, respectively) (Fig. 4). Only at 12 months after surgery, mTLT was less effective in IOP reduction in comparison to CW-TSCPC (WMD=-0.55, 95% CI -0.82 to -0.28) with no significant heterogeneity among the 3 included studies (I^2^ = 0.00%, P = 0.46).

#### Reduction in the number of antiglaucoma medications

**Fig. 5:**
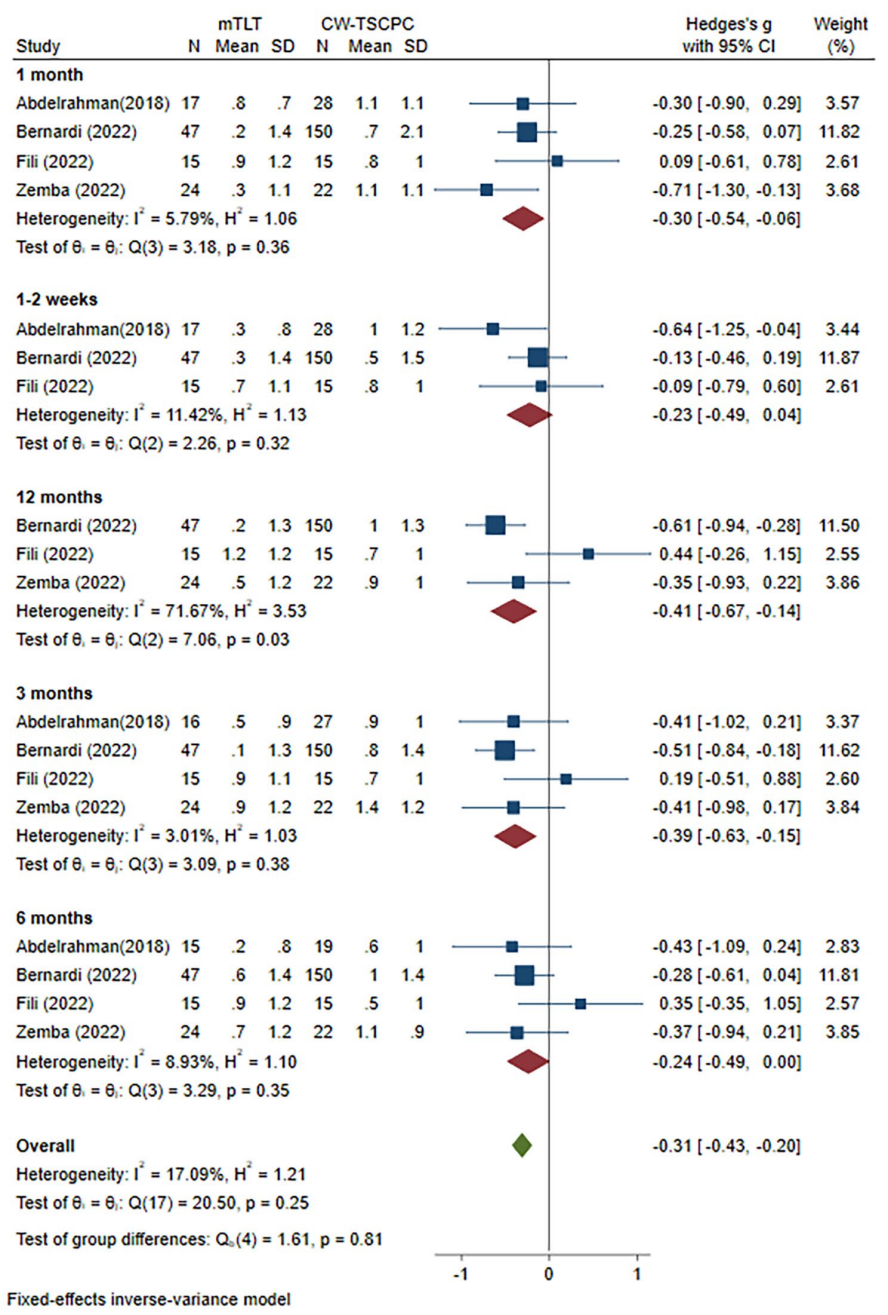
Meta-analysis of mTLT compared to CW-TSCPC in NOAM reduction at various follow-up visits. A total of 103 patients treated with mTLT and 215 patients treated with CW-TSCPC were included. The NOAM reduction was lower at 1 month (WMD=-0.30, 95% CI -0.54 to -0.06), and 3 months (WMD=-0.39, 95% CI -0.63 to -0.15) in mTLT, while there was no significant change at 1–2 weeks, and 6 months

We also analysed the changes in NOAM in 4 publications that reported complete data on NOAM at each follow-up visit after surgery by mean and standard deviation. We first assessed the NOAM changes before and after mTLT produce at various visits. The results demonstrated a decrease in NOAM till 6 months after treatment and no significant change at 12 months (WMD=-0.45, 95% CI -0.91 to -0.01) **(Supplement** Fig. 2**)**. In addition, there was no significant difference in the baseline NOAM (WMD=-0.06, 95% CI -0.30 to 0.18; heterogeneity: I^2^ = 0.00%, P = 0.62) between these two modalities of laser treatment **(Supplement** Fig. 3**)**. The burden of medication was lower after CW-TSCPC treatment at 1 month (WMD=-0.30, 95% CI -0.54 to 0.06), 3 months (WMD=-0.39, 95% CI -0.64 to 0.14), and 12 months (WMD=-0.41, 95% CI -0.67 to 0.14)with low heterogeneities at 1 month and 3 months (I^2^ = 5.79%, P = 0.36; I^2^ = 3.01%, P = 0.38, respectively) but high heterogeneity at 12 months (I^2^ = 71.67%, P = 0.03). In contrast, no difference was found at 1–2 weeks (WMD=-0.23, 95% CI -0.49 to 0.04), 6 months (WMD=-0.24, 95% CI -0.49 to 0.00), but there was low heterogeneity (I^2^ = 11.42%, P = 0.32; I^2^ = 8.93%, P = 0.35, respectively) **(**Fig. 5**)**. Collectively, mTLT treatment seemed to be inferior to the CW-TSCPC procedure in the NOAM reduction.

#### The retreatment rates

**Fig. 6:**
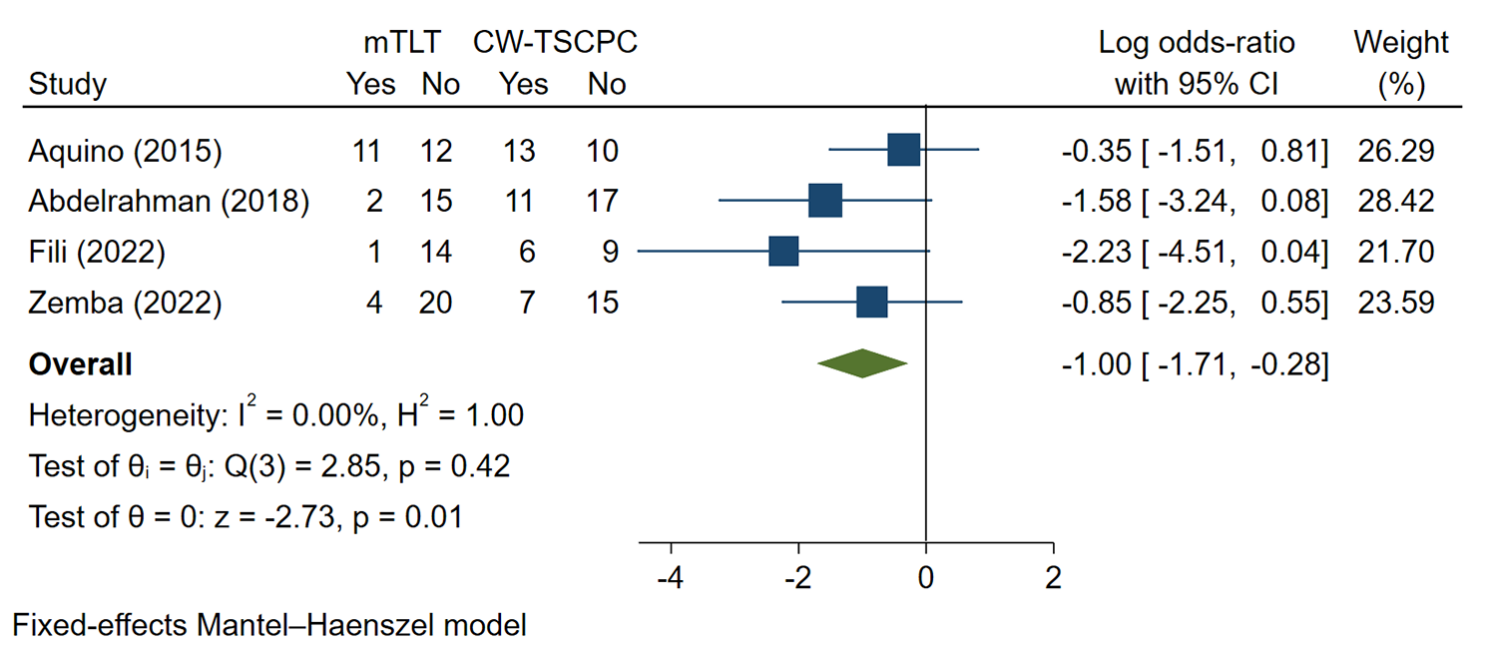
Meta-analysis of mTLT compared to CW-TSCPC in retreatment rate after the first treatment. A total of 80 patients underwent mTLT, and 88 patients treated with CW-TSCPC were included. The retreatment rate was lower in mTLT (Log OR=-1.00, 95% CI -1.71 to -0.28)

A total of 4 studies were included for the analysis of retreatment rates. The mTLT group exhibited a lower risk of surgical retreatment than the CW-TSCPC group (Log OR=-1.00, 95% CI -1.71 to -0.28), with low heterogeneity (I2 = 0.00%, P = 0.01) **(**Fig. 6**)**.

### Therapeutic safety assessments

#### Complications

**Fig. 7:**
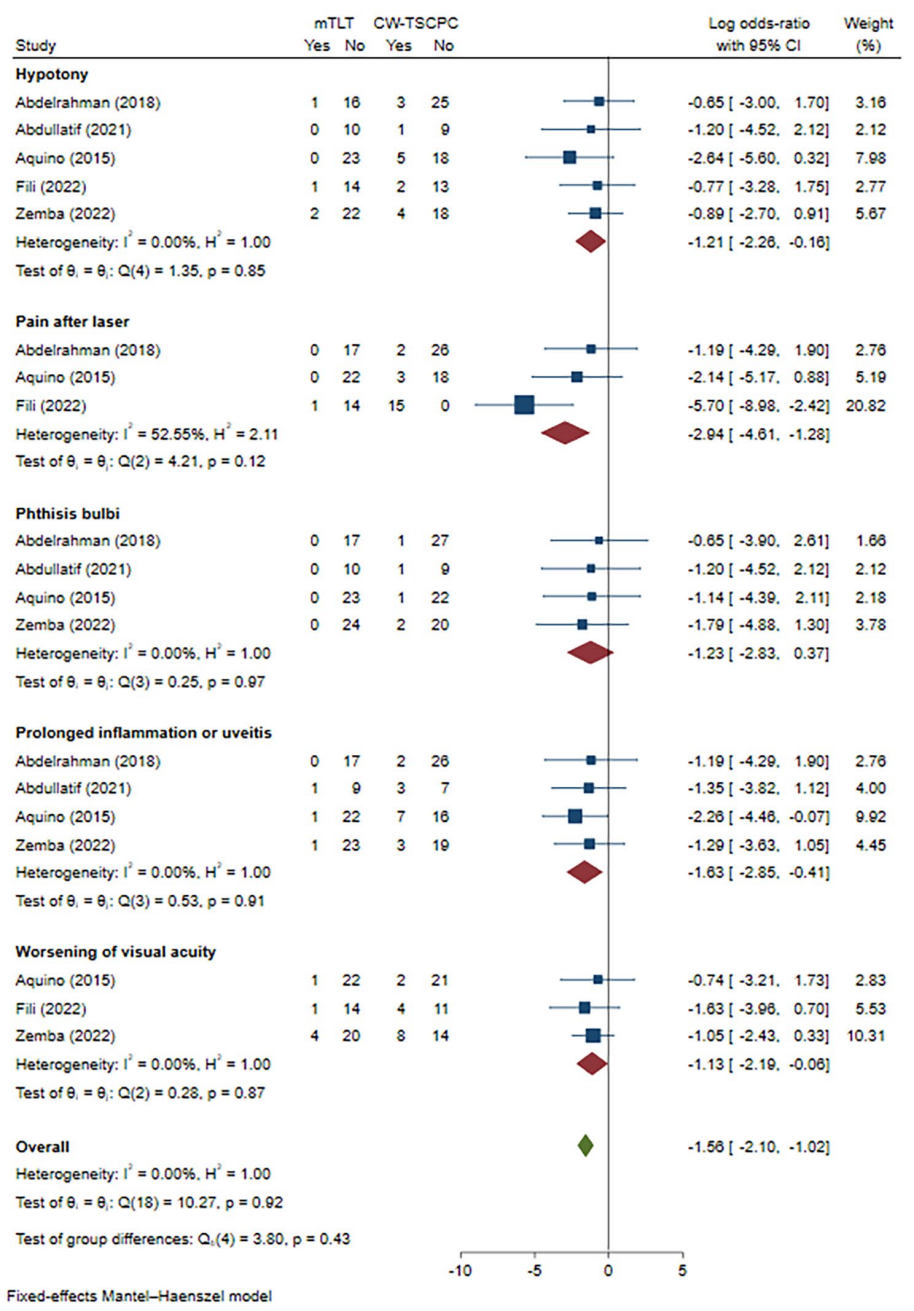
Meta-analysis of mTLT compared to CW-TSCPC in complications. A total of 89 patients underwent mTLT and 98 patients treated with CW-TSCPC were included. The risk of hypotony (Log OR=-1.21, 95% CI -2.26 to -0.16), prolonged inflammation or uveitis (Log OR=-1.63, 95% CI -2.85 to -0.41), and worsening of visual acuity (Log OR=-1.13, 95% CI -2.19 to 0.06) was lower after mTLT. No significant change was noted in terms of phthisis bulbi

To assess the safety of mTLT treatment, we compared the complications between mTLT and CW-TSCPC in 5 studies. The complications included hypotony, pain after laser, phthisis bulbi, prolonged inflammation or uveitis, and worsening of visual acuity (heterogeneity: I^2^ = 0.00%, P = 0.85; I^2^ = 52.55%, P = 0.12; I^2^ = 0.00, P = 0.97; I^2^ = 0.00%, P = 0.91; I^2^ = 0.00%, P = 0.87, respectively). There was no statistical significance in phthisis bulbi (Log OR=-1.23, 95% CI -2.83 to 0.37). Hypotony (Log OR=-1.21, 95% CI -2.26 to -0.16), pain after laser (Log OR=-2.94, 95% CI -4.61 to -1.28), prolonged inflammation or uveitis (Log OR=-1.63, 95% CI -2.85 to -0.41), and worsening of visual acuity (Log OR=-1.13, 95% CI -2.19 to -0.06) occurred less frequently in the mTLT group **(**Fig. 7**)**.

### Publication bias

Egger’s regression test and Begg’s test were applied to test publication bias. Publication bias was only found in medication comparison before and after mTLT (Table 3). There is a significant difference in the results before and after trim-and-fill analysis, indicating that publication bias could potentially affect the stability of the study results (**Supplement** Fig. 4).

**Table 3:** Publication bias

	Publication bias (Egger regresion test/ Begg’s test) (*P* value)
**before and after mTLT**	
IOP	0.1922 / 0.1696
Medication	0.0003 / 0.0000
**mTLT vs. CW-TSCPC**	
IOP before treatment	0.1046 / 1.0000
IOP after treatment	0.5499 / 0.9102
Medication before treatment	0.4192 / 0.3082
Medication after treatment	0.1232 / 0.0956
Retreatment	0.1015 / 0.0894
Complications	0.3020 / 0.4404

## Discussion

The microPulse P3 ® Delivery Device (IRIDEX Corporation, Mountain View, CA, USA) contains a customized contact MP3 probe and a Cyclo G6 Glaucoma Laser System that emits 810 nm infrared radiation [8]. It delivers repetitive micropulses of diode laser energy with a standard 31.3% duty cycle, a short working cycle ‘on’ and a longer rest cycle ‘off’. This special modality could make melanin in the pigmented ciliary epithelium absorb the energy from the 810 nm laser and transmit the energy to the surrounding tissue during the “on” cycle, and gradually cool the ciliary body during the “off” cycle, resulting in reduced the cyclodestructive damage histologically [8, 9], potentially achieving fewer complications clinically. Despite the confirmed efficacy of CW-TSCPC, excessive laser energy may cause collateral damage to the surrounding tissue, leading to persistent hypotony, intraocular inflammation, hyphema, or phthisis bulbi as clinical manifestations. The definite mechanism in CW-TSCPC is to suppress aqueous humour production by ciliary body destruction, while that in mTLT remains unrevealed. The possible mechanisms have been hypothesized.

During the ‘on’ cycle, the pigmented epithelium of the ciliary body absorbs the energy from the 810-nm laser and subsequently transmits it to the surrounding tissue. During the ‘off’ cycle, the pigmented ciliary epithelium, non-pigmented epithelium, and ciliary stroma can be cooled and protected from significant destruction as confirmed by macroscopic and histological evaluation at a low energy level. However, partial disruption of nonpigmented and pigmented ciliary epithelium layers, and stromal destruction in the pars plicata were observed at higher levels of energy in normal porcine eyes. As a result, the limited temperature rise could reduce the aqueous humour production and cyclodestructive damage simultaneously. In addition, it has been reported that the depigmented areas in the pars plana, coagulation of collagen and destruction of ciliary body stroma in mTLT were milder than those in CW-TSCPC in the adult cadaveric eyes [10, 11]. These findings suggested that mTLT was associated with reduced aqueous humour production.

A thicker choroid could be noted in the responsive patients after mTLT treatment by optical coherence tomography angiography in some studies [12]. In addition, Nemoto et al. obtained the direct signal from the uveoscleral outflow tract by a fluorescent tracer in Dutch belted rabbits treated with mTLT. Moreover, they suggested that uveoscleral outflow was dependent on the laser settings [13]. The correlation between the increase in choroidal thickness and the IOP reduction was significant, which suggested that the uveoscleral outflow pathway might be one of the important mechanisms [12].

The third possible mechanism is the trabecular meshwork outflow. Ahn et al. examined the histologic changes in the pars plicata and ciliary muscle in normal porcine eyes at different energies and found that the trabecular meshwork became longer in length after mTLT treatment at 1 month postoperatively. In addition, the expression of α-smooth muscle actin (α-SMA) reflected that the activation of myofibroblasts gradually increased in stromal myofibroblasts of ciliary muscle with enhanced laser energy after mTLT treatment, meaning that one of the mechanisms of mTLT might be the trabecular meshwork outflow pathway by ciliary muscle contraction and scleral spur displacement and this effect was energy-dependent [11].

In this meta-analysis, mTLT was compared with CW-TSCPC in regard to IOP reduction, NOAM reduction, retreatment rate, and complications. mTLT seems to be superior in safety and retreatment. However, a relatively high level of heterogeneity among the included studies was present in the comparison of IOP reduction and NOAM reduction. The diversities in baseline patient characteristics across different studies might explain this discrepancy. The baseline IOP in mTLT was lower than that in CW-TSCPC, and glaucoma type, glaucoma stage, laser parameters, age, previous history, and race were dissimilar among studies. Only the baseline of NOAM had no significant difference.

The results of this meta-analysis provided evidence that mTLT could be recommended as a new approach for glaucoma treatment. Compared to CW-TSCPC, mTLT has more advantages. First, unlike ciliary body destruction, which suppress aqueous humour production in CW-TSCPC, mTLT only slightly destroys the ciliary body, and improves the aqueous humour outflow through the uveoscleral and trabecular meshwork outflow pathway. Second, with the superiority of safety, mTLT widens the indications, not just for advanced stage glaucoma patients. Third, the repetitive treatment with mTLT enables the performance of invasive surgery in the future especially for younger patients with high surgical failure rates. Our results showed that mTLT could significantly lower the IOP with or without medicine in 12 months. In our included studies, the mean IOP reduction was 23.9–63% at 6 months, and 23–31% at 12 months, while the success rate was 60–70% at 6 months, 33-87.5% at 12 months, and 52% at 18 months. However, the efficacy might be influenced by several factors. First, the efficacy may be related to the type of glaucoma. After mTLT, all three mechanisms of IOP reduction might be activated, but with the differences in mechanisms of IOP elevation among various types of glaucoma, the effects of IOP lowering remain different to varying degrees [14–17]. As most studies have reported, neovascular glaucoma seemed to be less effective in controlling IOP by mTLT [14, 18]. However, which type of glaucoma could achieve a better result requires more evidence in the future. Second, the higher parameters of laser settings and durations are considered to have a more powerful IOP lowering effect in cyclophotocoagulation treatment, as a cause of a better efficacy [19]. Third, the age affects the effectiveness. The variations in ciliary body position leading to laser failure to the ciliary body and the excessive repair capacity of non-pigmented epithelial cells in children could result in a lower success rate than that in adults [20–22]. Fourth, the various sensitivities to mTLT treatment might also depend on the base condition including prior surgical history and baseline IOP [23–25]. Moreover, for different races, the various contents of pigment cells might also lead to different efficacies [26–29].Commonly, the antiglaucoma medications are mandatory postoperatively because of the delayed IOP-controlling effect in mTLT. As reported, most of the patients could reduce their medical burden but could not be completely freed from it. The number of medications could be reduced to a certain extent, but the decrease gradually decreased over time. We hypothesized that the progressive repairment in mild histological destruction [10] of mTLT and gradual diminishment in the reversible effect of increased aqueous humour outflow might contribute to this outcome. In this meta-analysis, ocular hypertension and several types of glaucoma, such as primary open-angle, primary angle-closure, neovascular, pseudoexfoliative, congenital, uveitis, pigmentary, postkeratoplasty, postvitrectomy, aphakic, and iridocorneal endothelial (ICE) syndrome glaucoma at various stages were included. In traditional treatment, only those who suffer from refractory glaucoma at the end stage are suggested to undergo cyclodestructive procedure as the last resort due to the high risks of complications. These limitations promote the development of cyclodestructive modalities. The superiority of fewer side effects related to mTLT produce drives its application in an increasing number of patients. Venkata et al. first reported the implementation of mTLT in patients with good central vision BCVA of ≥ 20/60. After treatment, their BCVA had no significant change, with a mean IOP reduction of 40.2% and a mean antiglaucoma medication decline of ≥ 1 from baseline to 12 months postoperatively, laying a foundation for application in early-stage glaucoma [30]. Subsequently, Abdullatif et al. reported a 23.9% IOP reduction and 60% success rates in the nonrefractory medically uncontrolled patients, providing evidence for the reduced burden of anti-glaucoma medication in mTLT [28] at nonrefractory glaucoma. Since the opaque cornea increases the surgical difficulty in the postkeratoplasty refractory glaucoma and filtration surgery usually leads to high rates of graft failure, Zemba et al. tried to apply mTLT to patients after penetrating keratoplasty. They obtained a satisfactory result, in which the success rate was 76.9%, and the median IOP dropped from baseline 29 mmHg to 18 mmHg at 12 months postoperatively [31]. Collectively, mTLT treatment has been gradually applied to various types of patients with acceptable consequences.

In fact, those who are not candidates for incisional glaucoma surgery or reject incisional surgery, those who have a history of incisional surgery, those who are refractory glaucoma patients, and those who wish to decrease the medical burden with a controlled IOP could be taken into consideration for mTLT treatment [15, 16, 23–25, 32–34]. However, mTLT is not suitable for every individual. For patients with scleral thinning secondary to glaucoma surgeries or rheumatoid arthritis, the area of scleral thinning should be avoided, and the energy should be limited to lower the energy transmission. Moreover, mTLT treatment is generally not recommended in patients with > 1 clock position of scleral thinning among the publications [35]. In addition, anti-inflammatory therapy is central to those who are at high risk of postoperative inflammation after treatment [36, 37]. The standard protocol for mTLT treatment is a laser setting of 2000 mW and a duty cycle of 31.3%, while the 3 and 9 clock positions are not treated [25]. Actually, the parameter settings were mostly dependent on surgeons’ clinical experience and patients’ preoperative IOP level, medical and surgical history and so on. Tekeli et al. tried to compare the outcomes between different laser durations (160 and 240 s) in 76 patients with good baseline visual acuity. The visual acuity and visual field values remained statistically unchanged from baseline to endpoints in two groups. However, the retreatment rates were higher in 160 s group (37.6%) than 240 s group (15.9%) at 12 months [19]. On the other hand, Niten Vig et al. lowered the energy (a duration of 80–90 s, a laser setting of 2000 mW and a duty cycle of 31.3%) for refractory glaucoma, and the success rates were 75.9% at 1 month, 79.3% at 3 months and 58.6% at 6 months [38]. It seemed that a longer duration could result in better IOP control and a lower retreatment rate. However, the balance between short-term and long-term efficacy and safety also needs to be taken into consideration during mTLT treatment. Some patients needed repeated mTLT treatment owing to uncontrolled IOP or failure to achieve target IOP, and the success rates gradually decreased over time in the included articles. Some studies showed that retreatment seemed to be helpful in further IOP control. In 2019, Lim et al. reported the results of repeated mTLT procedure in 43 Asian patients with refractory glaucoma. All of them received ≥ 2 episodes of mTLT with a mean IOP of 35.2 ± 11.0 mmHg preoperatively, 31.8 ± 13.2 mmHg at 3 years, and 27.1 ± 13.8 mmHg at the latest follow-up. Moreover, the average number of medications was reduced from 3.3 ± 0.9 preoperatively to 2.8 ± 1.3 at the final follow-up with few complications, including prolonged hypotony in 3 eyes (7.0%) and phthisis bulbi in 2 eyes (4.7%)[39]. In addition, repeated mTLT treatment could increase the success rates from 36.5 to 58% [20] and from 65 to 80% at the last follow-up [33]. However, the high level of baseline IOP, a younger age, and a prior CW-TSCPC might be associated with a mildly elevated risk of failure and complications [20, 27], while neither sex, race, visual field, type of glaucoma, nor a glaucoma family history might contribute to treatment failure [27, 40]. These findings demonstrated that glaucoma patients could benefit from repeated mTLT treatment without causing more side effects.

In our results, mTLT seems to be low-risk but with fewer included publications. The complication rates varied between the studies mostly because of the diversity in energy settings. It could be believed that a higher level of energy is associated with more severe and prolonged complications. Worsening of visual acuity, and phthisis bulbi are rare and considered severe complications. However, the decrease in sight might be mainly caused by aggravated cataracts [30], which could be resolved through cataract surgery. Common complications, such as mydriasis, superficial punctate keratitis [20], anterior chamber inflammation, hypotony, and pain seldom need more surgical intervention and can be recovered spontaneously or by medicine. Rare complications (IOP spikes, hyphema [28], scleral thinning [41], choroidal detachment [28], neurotrophic keratitis [42], and suprachoroidal haemorrhage [43]) can be alleviated after appropriate treatment. These findings reflected that mTLT treatment seems to be a safe option for glaucoma.

### Strengths and limitations

Our study involved a thorough search of the literature and was the first meta-analysis to compare the efficacy and safety of mTLT and CW-TSCPC in glaucoma treatment. Previous reviews only reported the results of individual studies without conducting a systematic review of the evidence. Although our study showed that mTLT might have superior safety and a lower rate of retreat than CW-TSCPC, there are several limitations in this meta-analysis. First, only 6 studies were included, of which only 2 were RCTs. Second, there were some differences in the baseline patient characteristics between the two groups. Third, the included patients suffering from various types of glaucoma at different stages underwent laser treatment with different settings and durations. Fourth, some different definitions of outcomes, such as success rate, which determined various criteria for retreatment made the comparison of efficacy more difficult. Fifth, the heterogeneity was high in IOP and NOAM reduction after treatment, and the source of this heterogeneity could not be explained. More RCTs with longer follow-up durations and larger sample sizes are needed to verify the efficacy and safety of mTLT in the future.

## Conclusion

In summary, our results showed that mTLT treatment could significantly lower the IOP until 12 months. However, the efficacy of IOP reduction is gradually diminished over time, and retreatment is necessary in some individuals. In addition, the retreatment rate in mTLT is likely to be lower than that in CW-TSCPC. Moreover, mTLT produce seems to be safer in short-term observation. Due to the limited numbers of included publications, our results need to be interpreted with cautioncritical to consideration.

## Electronic supplementary material

Below is the link to the electronic supplementary material.

Supplementary Material 1

Supplementary Material 2

**Supplement Figure 1**. Meta-analysis of mTLT comparing to CW-TSCPC in baseline IOP. A total of 113 patients treated with mTLT and 225 patients treated with CW-TSCPC was included. The baseline IOP in CW-TSCPC group was higher than that in mTLT group (WMD=-0.31, 95% CI -0.55 to -0.08). **Figure 2**. Meta-analysis of post- and pre-operative NOAM in mTLT at various visits. A total of 103 patients treated with mTLT was included. The IOP significantly reduced at 1-2 weeks, 3, and 6 months (WMD=-0.35, 95% CI -0.66 to -0.04; WMD=-0.51, 95% CI -0.91 to -0.11; WMD=-0.59, 95% CI -0.77 to -0.22, respectively?after mTLT treatment. **Figure 3**. Meta-analysis of mTLT comparing to CW-TSCPC in baseline NOAM. A total of 103 patients treated with mTLT and 215 patients treated with CW-TSCPC was included. No significant difference was observed between these two modalities of laser treatment (WMD=-0.06, 95% CI -0.30 to 0.18). **Figure 4**. Filled funnel plot of NOAM before and after mTLT.

## Data Availability

All data needed to evaluate the conclusions in the paper are present in the paper or the Supplementary Materials.
