# Supplementary material for: The efficacy and safety of micropulse transscleral laser treatment in glaucoma: a systematic review and meta-analysis
Source: BMC Ophthalmol. 2023 Jun 12;23:263. doi: 10.1186/s12886-023-03017-w (PMC10259047; doi:10.1186/s12886-023-03017-w)
Supplement: Supplementary file 2 — Supplementary Material 2 [file 12886_2023_3017_MOESM2_ESM.pdf]

Database: PubMed
Data: from 2000-01-01 to 2022-07-29
Records: 634

((((((((((((((((((((Thermocoagulation, Laser[Title/Abstract]) OR (Coagulation, Laser[Title/Abstract])) OR (Coagulations, Laser[Title/Abstract])) OR (Laser Coagulations[Title/Abstract])) OR (Laser Thermocoagulation[Title/Abstract])) OR (Laser Thermocoagulations[Title/Abstract])) OR (Thermocoagulations, Laser[Title/Abstract])) OR (Transscleral cyclophotocoagulation with MicroPulse laser[Title/Abstract])) OR (MicroPulse™ transscleral cyclophotocoagulation[Title/Abstract])) OR (Micropulse Transscleral Diode Cyclophotocoagulation[Title/Abstract])) OR (Micropulse Transscleral Diode Laser Cyclophotocoagulation[Title/Abstract])) OR (MicroPulse® transscleral laser therapy[Title/Abstract])) OR (micropulse cyclophotocoagulation[Title/Abstract])) OR (micropulse transscleral laser cyclophotocoagulation[Title/Abstract])) OR (Micropulse Diode Transscleral Cyclophotocoagulation[Title/Abstract])) OR (Micropulse Laser Transscleral Cyclophotocoagulation[Title/Abstract])) OR (Micropulse Trans-scleral Cyclophotocoagulation[Title/Abstract])) OR (Micropulse trans-scleral diode laser cyclophotocoagulation[Title/Abstract])) OR (Micropulse Transscleral Laser Treatment[Title/Abstract])) OR (micro-pulse transscleral cyclophotocoagulation[Title/Abstract]) OR (Laser Coagulation[mh]) AND ((Glaucomas[Title/Abstract]) OR (Glaucoma[mh]))

Database: Cochrane library
Data: from 2000-01-01 to 2022-07-29
Records: 115

(((((((((((((((((((('Thermocoagulation, Laser':ab,ti) OR ('Coagulation, Laser':ab,ti)) OR ('Coagulations, Laser':ab,ti)) OR ('Laser Coagulations':ab,ti)) OR ('Laser Thermocoagulation':ab,ti)) OR ('Laser Thermocoagulations':ab,ti)) OR ('Thermocoagulations, Laser':ab,ti)) OR ('Transscleral cyclophotocoagulation with MicroPulse laser':ab,ti)) OR ('MicroPulse™ transscleral cyclophotocoagulation':ab,ti)) OR ('Micropulse Transscleral Diode Cyclophotocoagulation':ab,ti)) OR ('Micropulse Transscleral Diode Laser Cyclophotocoagulation':ab,ti)) OR ('MicroPulse® transscleral laser therapy':ab,ti)) OR ('Micropulse cyclophotocoagulation':ab,ti)) OR ('Micropulse transscleral laser cyclophotocoagulation':ab,ti)) OR ('Micropulse Diode Transscleral Cyclophotocoagulation':ab,ti)) OR ('Micropulse Laser Transscleral Cyclophotocoagulation':ab,ti)) OR ('Micropulse Trans-scleral Cyclophotocoagulation':ab,ti)) OR ('Micropulse trans-scleral diode laser cyclophotocoagulation':ab,ti)) OR ('Micropulse Transscleral Laser Treatment':ab,ti)) OR ('micro-pulse transscleral cyclophotocoagulation':ab,ti) OR ('Laser Coagulation' [mh]) AND ('Glaucoma' [mh] OR 'Glaucoma':ab,ti)

Database: Embase
Data: from 2000-01-01 to 2022-07-29
Records: 2257

((((((((((((((((((((('Thermocoagulation, Laser':ab,ti) OR ('Coagulation, Laser':ab,ti)) OR ('Coagulations, Laser':ab,ti)) OR ('Laser Coagulations':ab,ti)) OR ('Laser Thermocoagulation':ab,ti)) OR ('Laser Thermocoagulations':ab,ti)) OR ('Thermocoagulations, Laser':ab,ti)) OR ('Transscleral cyclophotocoagulation with MicroPulse laser':ab,ti)) OR ('MicroPulse™ transscleral cyclophotocoagulation':ab,ti)) OR ('Micropulse Transscleral Diode Cyclophotocoagulation':ab,ti)) OR ('Micropulse Transscleral Diode Laser Cyclophotocoagulation':ab,ti)) OR ('MicroPulse® transscleral laser therapy':ab,ti)) OR ('Micropulse cyclophotocoagulation':ab,ti)) OR ('Micropulse transscleral laser cyclophotocoagulation':ab,ti)) OR ('Micropulse Diode Transscleral Cyclophotocoagulation':ab,ti)) OR ('Micropulse Laser Transscleral Cyclophotocoagulation':ab,ti)) OR ('Micropulse Trans-scleral Cyclophotocoagulation':ab,ti)) OR ('Micropulse trans-scleral diode laser cyclophotocoagulation':ab,ti)) OR ('Micropulse Transscleral Laser Treatment':ab,ti)) OR ('micro-pulse transscleral cyclophotocoagulation':ab,ti) OR ('Laser Coagulation' /exp) AND ('Glaucoma'/exp OR 'Glaucoma':ab,ti)
