# Supplementary material for: The efficacy and safety of micropulse transscleral laser treatment in glaucoma: a systematic review and meta-analysis
Source: BMC Ophthalmol. 2023 Jun 12;23:263. doi: 10.1186/s12886-023-03017-w (PMC10259047; doi:10.1186/s12886-023-03017-w)

Supplement

Figure 1. Meta-analysis of mTLT comparing to CW-TSCPC in baseline IOP. A total of 113 patients treated with mTLT and 225 patients treated with CW-TSCPC was included. The baseline IOP in CW-TSCPC group was higher than that in mTLT group (WMD=-0.31, 95% CI -0.55 to -0.08).

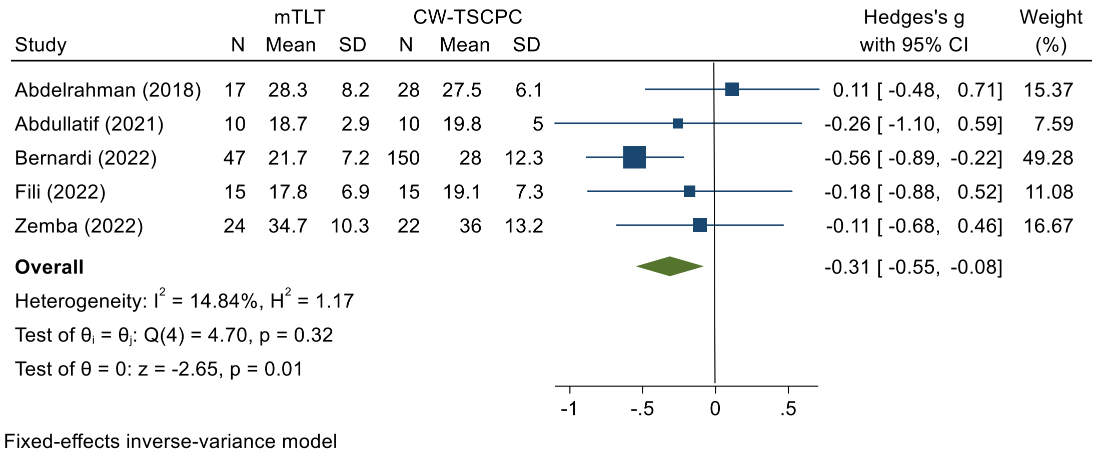

Figure 2. Meta-analysis of post- and pre-operative NOAM in mTLT at various visits. A total of 103 patients treated with mTLT was included. The IOP significantly reduced at 1-2 weeks, 3, and 6 months (WMD=-0.35, 95% CI -0.66 to -0.04; WMD=-0.51, 95% CI -0.91 to -0.11; WMD=-0.59, 95% CI -0.77 to -0.22, respectively）after mTLT treatment.

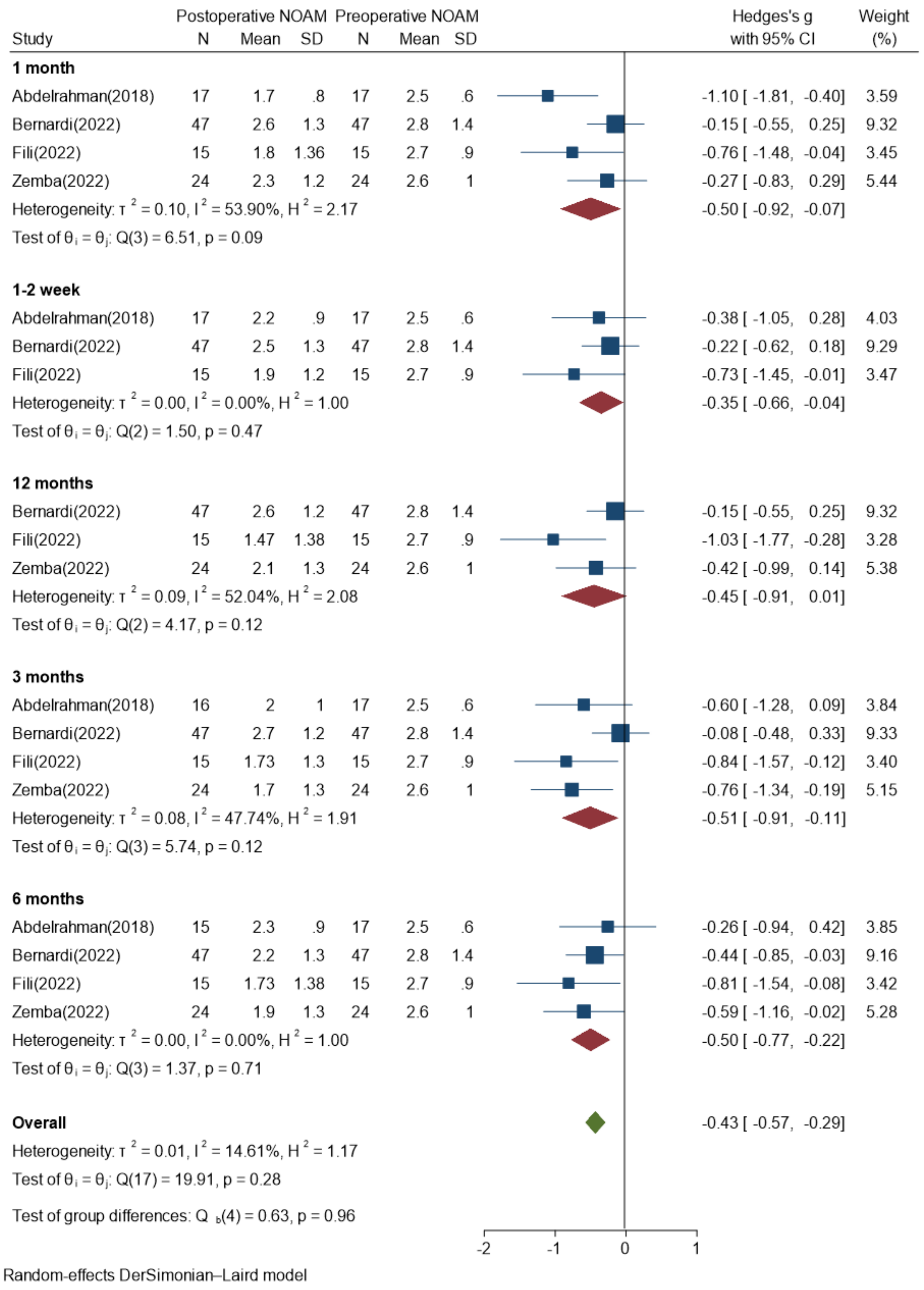

Figure 3. Meta-analysis of mTLT comparing to CW-TSCPC in baseline NOAM. A total of 103 patients treated with mTLT and 215 patients treated with CW-TSCPC was included. No significant difference was observed between these two modalities of laser treatment (WMD=-0.06, 95% CI -0.30 to 0.18).

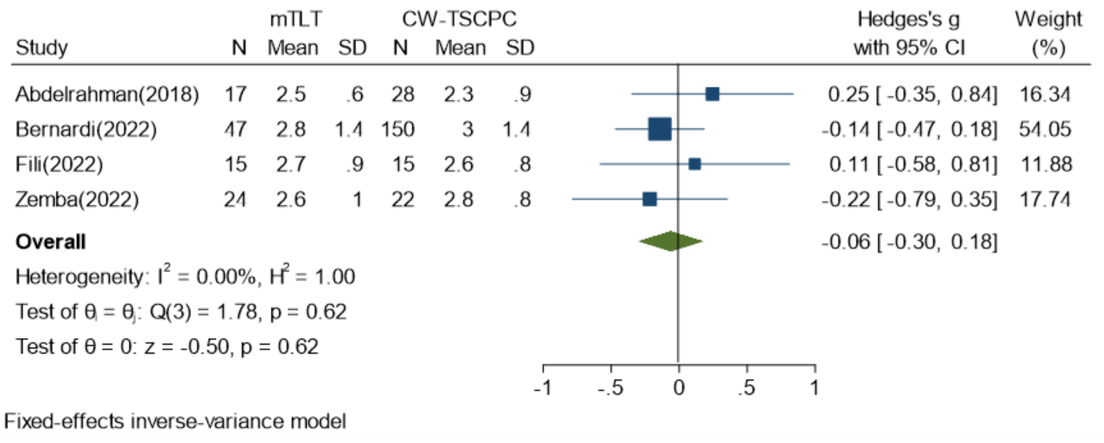

Figure 4. Filled funnel plot of NOAM before and after mTLT.

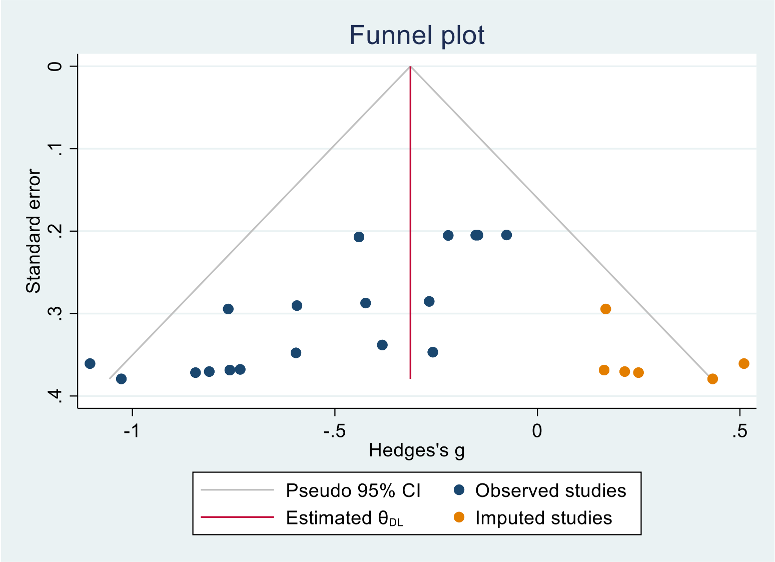

Supplement: Supplementary file 3 — Supplement Figure 1. Meta-analysis of mTLT comparing to CW-TSCPC in baseline IOP. A total of 113 patients treated with mTLT and 225 patients treated with CW-TSCPC was included. The baseline IOP in CW-TSCPC group was higher than that in mTLT group (WMD=-0.31, 95% CI -0.55 to -0.08). Figure 2. Meta-analysis of post- and pre-operative NOAM in mTLT at various visits. A total of 103 patients treated with mTLT was included. The IOP significantly reduced at 1-2 weeks, 3, and 6 months (WMD=-0.35, 95% CI -0.66 to -0.04; WMD=-0.51, 95% CI -0.91 to -0.11; WMD=-0.59, 95% CI -0.77 to -0.22, respectively?after mTLT treatment. Figure 3. Meta-analysis of mTLT comparing to CW-TSCPC in baseline NOAM. A total of 103 patients treated with mTLT and 215 patients treated with CW-TSCPC was included. No significant difference was observed between these two modalities of laser treatment (WMD=-0.06, 95% CI -0.30 to 0.18). Figure 4. Filled funnel plot of NOAM before and after mTLT. [file 12886_2023_3017_MOESM3_ESM.pdf]
